# Prognostic factors associated with improvements in patient-reported outcomes in idiopathic adhesive capsulitis

**DOI:** 10.1016/j.jseint.2022.12.007

**Published:** 2022-12-20

**Authors:** Paul V. Romeo, Aidan G. Papalia, Matthew G. Alben, Neil Gambhir, Dhruv Shankar, Andrew S. Bi, Joseph D. Zuckerman, Mandeep S. Virk

**Affiliations:** Division of Shoulder and Elbow Surgery, Department of Orthopedic Surgery, NYU Grossman School of Medicine, NYU Langone Orthopedic Hospital, NYU Langone Health, New York, NY, USA

**Keywords:** Adhesive capsulitis, Frozen shoulder, PROMIS, Upper extremity, Factors, Patient-reported outcomes

## Abstract

**Background:**

The purpose of this study was to identify prognostic factors that are associated with improvements in patient-reported outcomes measures (PROMs) related to upper extremity function and pain in those suffering from idiopathic adhesive capsulitis.

**Methods:**

All patients treated conservatively for primary idiopathic adhesive capsulitis were identified from our institutional database between 2019 and 2021. Exclusion criteria included any patients treated surgically, follow-up less than one year, or incomplete survey results. PROMs including Patient-Reported Outcomes Measurement Information System (PROMIS) Upper Extremity Computer Adaptive Test Version 2.0 (P-UE), Pain Interference (P-Interference), Pain Intensity (P-Intensity), and visual analog scale (VAS) pain scores. They were obtained at initial consultation and at one year to assess patient-perceived impact of their condition. Multiple linear and multivariable logistic regressions were performed to identify factors associated with improvement in patient-perceived pain and shoulder function using final PROM scores and difference in PROM scores from initial consultation. An independent *t*-test was used to compare baseline and one-year minimum follow-up PROMs. Odds ratios and their 95% confidence intervals were calculated for each factor; a *P* value of < .05 was considered statistically significant.

**Results:**

A total of 56 patients (40 females and 16 males) were enrolled in the study with an average age of 54.7 ± 7.7 years. A significant improvement (*P* < .001) was demonstrated at one-year minimum outcomes for P-UE, P-Interference, P-Intensity, and VAS scores. With respect to comorbid conditions, hypothyroidism [P-UE (β: 9.57, *P* = .006)] was associated with greater improvements in PROMs, while hyperlipidemia [P-UE (β: −4.13, *P* = .01) and P-Intensity (β: 2.40, *P* = .02)] and anxiety [P-UE (β: −4.13, *P* = .03)] were associated with poorer reported changes in PROMs. Female sex [P-UE (β: 4.03, *P* = .007) and P-Interference (β: −2.65, *P* = .04)] and employment in manual labor professions [P-Interference (β: −3.07, *P* = .01), P-Intensity (β: −2.92, *P* = .006), and VAS (β: −0.66, *P* = .03)] were associated with significantly better patient-perceived outcomes. Hispanic heritage was associated with higher reported changes of P-Intensity (β: 8.45, *P* = .004) and VAS (β: 2.65, *P* = .002).

**Conclusion:**

Patient-perceived improvements in PROMIS score during the natural history of adhesive capsulitis are likely multifactorial, with anxiety, hyperlipidemia, increased body mass index, and Hispanic heritage associated with reduced improvement in PROMIS scores.

Idiopathic adhesive capsulitis (IAC) or frozen shoulder is a common shoulder pathology affecting 2%-5% of the general population.^34^ IAC is characterized by pain and decreased range of motion (ROM) due to inflammation and fibrosis of the synovial capsule, classically evolving through 3 overlapping phases: the painful freezing stage, the stiff frozen stage, and the resolving thawing stage.^34^ While the etiology of primary IAC remains largely unknown, several associated risk factors associated with the diagnosis of IAC have been established including female gender, thyroid disorders, and diabetes.^9^^,^^20^^,^^23^^,^^30^^,^^36^ However, prognostic risk factors for successful nonoperative treatment of IAC are not as well defined.

IAC has a long natural history with substantial pain and limited shoulder function for several months, resulting in significant morbidity in those afflicted and a significant socioeconomic burden to the healthcare system.^2^^,^^11^ Furthermore, due to the idiopathic nature of the condition and a disabling, protracted course, patients often look for answers regarding their prognosis and recovery via nonsurgical treatment. Although IAC is a self-resolving condition, it is increasingly important to identify factors predictive of improved patient outcomes to guide clinical management and set patient expectations.^2^^,^^22^ Patient-reported outcome measures (PROMs) are crucial tools in measuring treatment efficacy and disease progression in this population but there exists a paucity in the literature of studies investigating factors associated with PROMs in IAC.^22^

The purpose of this study is to identify prognostic factors associated with improvements in PROMs in primary IAC, particularly patient-perceived upper extremity function and reductions in patient-perceived pain related to daily tasks. We hypothesize that patients’ perception of pain and their functional limitations are multifactorial, incorporating both modifiable and nonmodifiable factors.

## Methods

### Study ethics

An internal Institutional Review Board Approval was granted for this study with all subjects providing informed consent prior to enrollment (s20-00287).

### Study design and cohort selection

This was a retrospective review conducted on a prospectively enrolled database of consecutive patients treated for IAC using International Classification of Diseases 10 (M75.00, M75.01, and M75.02) codes between August 16, 2019 and January 1, 2021. Baseline and final follow-up PROMs were assessed using Patient-Reported Outcomes Measurement Information System (PROMIS) scores obtained by telephone, e-mail, or in person during office follow-up visits as per patient preference.

Subjects were enrolled in this study if they met the following inclusion criteria: (1) age of 18 years or more at time of initial consultation, (2) underwent nonsurgical treatment for idiopathic adhesive capsulitis, (3) minimum of one-year follow-up from initial to final consultation, (4) completed the required PROMs at initial consultation, and (5) were able to provide informed consent. Subjects were excluded from the study if they were (1) deceased or lost to follow-up, (2) had adhesive capsulitis from a secondary etiology (eg, surgery, trauma), (3) were unable to communicate in English, (4) underwent any surgery on the affected shoulder, or (5) were unable to provide informed consent or complete the study surveys.

### Diagnosis and treatment

All patients underwent plain radiographic imaging (anteroposterior, axillary, and scapular Y view) to rule out causes of secondary adhesive capsulitis (eg, glenohumeral arthritis). The diagnosis of adhesive capsulitis was made by the treating physician based on pertinent clinical history and physical examination. All patients presenting with shoulder pain and global loss of ROM (both active and passive) and radiographs showing no signs of arthritis or calcific tendinitis were diagnosed with adhesive capsulitis.

The natural history of adhesive capsulitis was explained to each patient and the benefits and risks for each treatment option were discussed in detail. However, treatment was guided through shared decision-making based on patient preference. Patients were offered nonsurgical treatments in the form of oral anti-inflammatory medications (over the counter or prescription strength nonsteroidal anti-inflammatory medications, oral steroids), home exercise program or outpatient-supervised physical therapy, and intra-articular steroid injection. The final determination for intra-articular steroid injection was made by the patient.

### Patient-reported outcomes and factors measured

Subjects’ active ROM was measured individually by 2 trained research fellows using a goniometer. In addition, subjects were required to complete PROMIS Upper Extremity Computer Adaptive Test Version 2.0 (P-UE), PROMIS Pain Interference (P-Interference), and PROMIS Pain Intensity (P-Intensity) and a visual analog pain scale (VAS) at initial consultation and one-year minimum follow-up. Normalized across a scale of 0-100 with a mean value of 50 (SD ± 10) in reference to the general United States population, PROMIS has been established to accurately assess a patient's perceived symptomatology across all facets of orthopedics.^4^^,^^7^^,^^19^^,^^22^^,^^31^ Using a combination of item response theory and computer adaptive testing, PROMIS can reliably capture patient outcomes in less questions as compared to legacy scores.^8^^,^^29^^,^^37^

PROMIS-based PROMs are derived from 1 of 4 domains (physical, mental, global, and social health), each of which is further divided into a multitude of separate subdomains. In respect to P-UE, P-Interference, and P-Intensity, each of these belong to the physical health domain which is further comprised of subdomains such as physical function, physical activity, pain, fatigue, and sleep disturbances. As patients initially presenting for IAC are typically limited in ROM with concomitant pain in their affected shoulder, these 3 PROMs allow for accurate assessment of the two most common complaints in those afflicted. Specifically, P-UE evaluates one’s ability to use their shoulder, while P-Interference and P-Intensity assess the limitations brought about by their shoulder pain and the severity of pain they are currently experiencing, respectively. While higher scores in P-UE indicate superior upper extremity function, lower scores in PROMIS pain instruments (P-Interference, P-Intensity) are indicative of a patient experiencing less pain.

To determine which factors may influence improvement in PROMs, we evaluated a multitude of factors including previously reported risk factors for primary IAC.^1^, ^2^, ^3^^,^^6^^,^^12^^,^^14^^,^^15^^,^^18^^,^^20^^,^^21^^,^^24^^,^^25^^,^^28^^,^^30^^,^^33^^,^^34^^,^^36^ Existing literature revealed prior trauma, HLA-B27 positivity, age more than 40 years, female sex, thyroid disease, obesity, and autoimmune diseases to be predisposing risk factors for developing IAC.^13^^,^^20^^,^^23^^,^^26^^,^^32^^,^^35^^,^^36^ We in turn evaluated age, gender, body mass index (BMI), PROMIS scores and ROM at initial consultation (flexion, internal rotation, and external rotation), concomitant medical conditions (hypertension, hyperlipidemia, hypothyroidism, diabetes, anxiety, and depression), smoking status, marital status, ethnicity, manual versus nonmanual labor, dominant arm involvement, number of corticosteroid injections received, and time from symptom onset to first visit as these factors could all be ascertained from patient medical records and were investigated for a possible impact on the investigated outcomes.

### Statistical methods

All statistical analysis was performed in R-studio version 4.0.3 (R Studio, Boston, MA, USA) and SAS Studio Version 9.4 (SAS Institute, Cary, NC, USA). An independent *t*-test was used to compare baseline and one-year minimum follow-up PROMs. Multiple linear and multivariable logistic regressions were conducted to determine which factors were associated with patients’ improvement in pain and function as determined by the final PROM score and change in PROM scores from initial to final visit. Stepwise selection was applied to each regression model to identify variables that were the strongest predictors of each outcome modeled. Regression coefficients (β) were calculated for each predictor selected for a given model and predictors with *P* < .05 were considered statistically significant.

A post hoc analysis was performed using G∗Power version 3.1.9.7 (Heinrich Heine Universität, Düsseldorf, Germany) to determine achieved power on multivariable regression analysis. Our analysis was performed using postoperative PROMIS-UE as the outcome and the aforementioned factors as independent variables in a mixed multivariable regression. Using the partial R2 of 0.58, the effect size was determined to be 1.38. Using an F-test family parameter for multivariable regression models, the calculated effect size of 1.38, an alpha error probability of 0.05, and a total sample size of 57 patients, the achieved power for our analysis was determined to be 0.98.

## Results

### Cohort characteristics

A total of 56 patients were included in the study with an average age of 54.7 ± 7.7 (range 43-74 years) years. There were 40 females (71.4%) and 16 males (28.6%). Mean BMI was 27.1 (range 18.97-44.61). Throughout the course of treatment, 39/56 (69.6%) patients received a glenohumeral steroid injection with an average of 1.2 ± 0.4 (range 0-2) and the dominant arm was affected in 46.4% (26/56) of subjects. A complete list of patient demographics is provided in Table I.

**Table I:** Patient demographics.

	Overall (N = 56)		
Age		BMI	
Mean (SD)	54.7 (±7.7)	Mean (SD)	27.1 (±6.6)
Sex		Concomitant medical conditions	
Female	40 (71.4%)	Diabetes mellitus	12 (21.4%)
Male	16 (28.6%)	Hypertension	16 (28.6%)
Race		Hyperlipidemia	19 (33.9%)
Asian	9 (16.1%)	Hypothyroidism	2 (3.6%)
Black or African American	8 (14.3%)	Anxiety	7 (12.5%)
White	31 (57.4%)	Depression	7 (12.5%)
Unknown/Declined to report	8 (14.3%)	Manual laborer	13 (23.2%)
Hispanic ethnicity	4 (7.1%)	Nonmanual laborer	43 (76.8%)
Smoking Status		Corticosteroid Injection	39 (69.6%)
Never	29 (51.8%)	Mean (SD)	1.2 (±0.4)
Current	5 (8.9%)	Preoperative Range of Motion	
Former	22 (39.3%)	Flexion (SD)	119 (±31)
Affected Shoulder		External Rotation (SD)	40 (±17)
Dominant	26 (46.4%)	Internal Rotation∗ (SD)	3 (±1)
Left	28 (50%)	Baseline PROMIS Scores	
Right	28 (50%)	Upper Extremity	34.6 (±9.1)
Bilateral	0 (0%)	Pain Interference	58.1 (±7.1)
Time From Symptom Onset to First Visit		Pain Intensity	51.4 (±6.2)
Mean Days (sd)	104.1 (±99.3)	Baseline VAS (SD)	6.4 (±2.2)
		Time Between First Visit and Final Survey	
		Mean Days (SD)	528.8 (±128.9)

*SD*, standard deviation; *BMI*, body mass index; *PROMIS*, Patient Reported Outcome Measure Instrument Survey; *VAS*, visual analog scale.

∗ Internal rotation reported as per the standardized surgeon measure of active internal rotation scale by Mollon et al.

### Prognostic factors of patient-reported outcomes at final follow-up

Comparison of baseline PROM scores with one-year minimum follow-up outcomes can be seen in Table II. A significant improvement was found when comparing these 2 time points for each PROM: P-UE (34.6-46.4, *P* < .001), P-Interference (58.1-47.0, *P* < .001), P-Intensity (51.4-36.9, *P* < .001), and VAS (6.4-1.6, *P* < .001). Factors associated with changes in final PROM values can be seen in Table III. Furthermore, factors with an associated impact on the change in PROM score from baseline are demonstrated in Table IV.

**Table II:** Patient-reported outcomes measure.

PROM test	PROM score ± standard deviation	Significance
Baseline PROMIS UE	34.6 (±9.1)	*P* < .001
Follow-up PROMIS UE	46.4 (±12.5)	
Baseline PROMIS P-Interference	58.1 (±7.1)	*P* < .001
Follow-up PROMIS P-Interference	47.0 (±9.9)	
Baseline PROMIS P-Intensity	51.4 (±6.2)	*P* < .001
Follow-up PROMIS P-Intensity	36.9 (±8.5)	
Baseline VAS	6.4 (±2.2)	*P* < .001
Follow-up VAS	1.6 (±2.4)	

*PROMIS UE*, Patient-Reported Outcomes Measurement Information System: Upper Extremity; *PROMIS P-Interference*, Patient-Reported Outcomes Measurement Information System: Pain Interference; *PROMIS P-Intensity*, Patient-Reported Outcomes Measurement Information System: Pain Intensity; *VAS*, visual analog scale.

**Table III:** Risk factors associated with changes in final PROMIS score.

PROMIS test	Predictor	Beta coefficient (β)	*P* value
PROMIS UE Follow-up Score	UE score initial visit	0.29	.048
	BMI	−0.49	.04
	Hyperlipidemia	−4.13	.01
	Anxiety	−4.19	.03
	Hypothyroidism	9.57	.006
	Female	4.03	.007
PROMIS P-Interference Follow-up Score	BMI	0.52	.004
	Dominant side involved	2.30	.05
	Female	−2.67	.03
	Manual labor	−3.07	.01
PROMIS P-Intensity Follow-up Score	BMI	0.41	.01
	Dominant side involved	2.07	.05
	Manual labor	−2.92	.006
VAS Follow-up Score	BMI	0.11	.02
	Dominant side involved	0.79	.01
	Manual labor	−0.66	.03

*BMI*, body mass index; *PROMIS UE*, Patient-Reported Outcomes Measurement Information System Upper Extremity; *PROMIS P-Interference*, Patient-Reported Outcomes Measurement Information System: Pain Interference; *PROMIS P-Intensity*, Patient-Reported Outcomes Measurement Information System: Pain Intensity; *UE*, upper extremity; *VAS*, visual analog scale.

**Table IV:** Risk factors associated with changes in the degree of change of PROMIS scores.

PROMIS test	Predicator	Beta coefficient (β)	*P* value
PROMIS UE Change in Score	UE score Initial Visit	−0.71	<.001∗
	BMI	−0.49	.04∗
	Hyperlipidemia	−4.13	.01∗
	Anxiety	−4.13	.03∗
	Hypothyroidism	9.57	.006∗
	Female	4.03	.007∗
PROMIS P-Interference Change in Score	P-Interference initial visit	−0.84	<.001∗
	BMI	0.59	.002∗
	Female	−2.65	.04∗
	Manual labor	−2.64	.03∗
PROMIS P-Intensity Change in Score	P-Intensity initial visit	−0.78	<.001∗
	Hyperlipidemia	2.40	.02∗
	Hispanic	8.45	.004∗
	Manual labor	−2.25	.03∗
VAS Change in Score	VAS initial visit	−0.91	<.001∗
	Hyperlipidemia	1.04	<.001∗
	Hispanic	2.65	.002∗

*BMI*, body mass index; *PROMIS UE*, Patient-Reported Outcomes Measurement Information System Upper Extremity; *PROMIS P-Interference*, Patient-Reported Outcomes Measurement Information System: Pain Interference; *PROMIS P-Intensity*, Patient-Reported Outcomes Measurement Information System: Pain Intensity; *UE*, upper extremity; *VAS*, visual analog scale.

∗ Significant values.

Higher BMI was associated with greater patient P-Intensity (β: 0.41, *P* = .01), P-Interference (β: 0.52, *P* = .004), VAS score (β: 0.11, *P* = .02), and a lower P-UE score (β: −0.49, *P* = .04). Dominant side involvement was also shown to increase patient perception of interference to daily tasks and pain level [P-Interference (β: 2.30, *P* = .05), P-Intensity (β: 2.07, *P* = .05), VAS (β: 0.79, *P* = .01)]. Conversely, manual labor professions were found to be a protective factor, demonstrating reduced perception of pain intensity and interference [P-interference (β: −3.07, *P* = .01), P-Intensity (β: −2.92, *P* = .006), and VAS (β: −0.66, *P* = .03)]. Similarly, female gender was associated with a reduction in P-Interference (β: −2.67, *P* = .03) and an increase in P-UE (β: 4.03, *P* = .007) scores. After accounting for confounding effects, higher P-UE baseline scores (β: 0.29, *P* = .048) and history of hypothyroidism (β: 9.57, *P* = .006) were associated with more favorable outcomes, while history of hyperlipidemia (β: −4.13, *P* = .01) and anxiety (β: −4.19, *P* = .03) were associated with worse outcomes (Table III). Age, preoperative ROM, diabetes, depression, smoking status, marital status, race, and number of corticosteroid injections received did not demonstrate a significant impact associated with reported final score outcomes.

### Prognostic factors associated with improvement in patient-reported outcomes from baseline

We evaluated the impact that established risk factors had on the overall change in a patient’s PROMIS scores from initial evaluation to final follow-up. As a factor, baseline PROMIS scores were determined to be a significant predictor of the magnitude of change for final follow-up PROMs [P-UE (β: −0.71, *P* < .001), P-Interference (β: −0.84, *P* < .001), P-Intensity (β: −0.78, *P* < .001), and VAS (β: −0.91, *P* < .001)]. Hyperlipidemia demonstrated a significant detrimental impact on the change of multiple PROM scores [P-UE (β: −4.13, *P* = .01), P-Intensity (β: 2.40, *P* = .02), and VAS (β: 1.04, *P* < .001)]. Similarly, BMI demonstrated a negative impact on the magnitude of change in P-UE (β: −0.49, *P* = .04) and P-Interference score (β: 0.59, *P* = .002). As compared to other races, Hispanic patients demonstrated a significantly smaller magnitude of change in P-Intensity (β: 8.45, *P* = .004) and VAS scores (β: 2.65, *P* = .002) throughout the course of the study. Female sex demonstrated improvement in initial to final P-UE (β: 4.03, *P* = .007) and P-Interference scores (β: −2.65, *P* = .04). Similarly, hypothyroidism demonstrated a likewise association for P-UE (β: 9.57, *P* = .006) (Tables III and IV). Age, preoperative ROM, depression, diabetes, hypertension, smoking status, marital status, and number of corticosteroid injections received did not demonstrate significant impact associations with preoperative to postoperative outcomes.

## Discussion

In our study, we found one-year follow-up PROM scores and the difference from their baseline scores are influenced by a variety of patient-related factors. Notably, increased BMI, hyperlipidemia, and dominant side involvement were associated with a decrease in shoulder functionality and an increase in shoulder pain, while involvement in manual labor professions, lower initial PROM scores, and female sex were associated with an increase in shoulder function and decrease in shoulder pain.

Knowledge of these modifiable and nonmodifiable factors and their associated effects provide physicians with a better understanding of a patient’s expected outcomes, thereby allowing physicians to better anticipate a patient’s perception and set more realistic treatment expectations. BMI, a modifiable risk factor, was shown in our study to be consistently associated with decreased shoulder function and increased pain. Evidenced by our study, patients with higher BMI experience worse function and increased pain once afflicted by IAC, but this population is also at an increased odds of suffering from IAC, with one study of 2190 patients finding an increased odds ratio of 1.26 (*P* < .001).^20^ However, these results are not universally agreed upon with another study of 87 patients showing decreased BMI to be associated with an increased overall risk of suffering from IAC (*P* = .02), more specifically a 3% increased risk of IAC for every kilogram of lower weight.^36^ Hyperlipidemia, another modifiable risk factor, has proven through our study to detrimentally impact shoulder function and increase pain. Similar to BMI, hyperlipidemia has previously been associated with an overall increased risk of suffering from IAC.^26^^,^^35^ A study of 28,748 records from The National Health Insurance Research Database of Taiwan showed hyperlipidemia to have a crude hazard ratio of 1.7 (95% confidence interval [CI] 1.61-1.79; *P* < .001) and an adjusted hazard ratio of 1.50 (95% CI, 1.41-1.59; *P* < .001).^35^ Further support of the correlative risk of IAC related to hyperlipidemia was confirmed through multivariate analysis performed by Lo et al on 1 million patients from the Taiwan National Health Insurance database. They found hyperlipidemia to be an independent risk factor associated with IAC, having a hazard ratio of 1.29 (95% CI 1.11-1.49, *P* < .001).^26^ Unfortunately, the external validity of these studies is limited due to the limited demographic diversity of the patient population.

In our study, baseline PROM scores, a nonmodifiable factor, proved to be a risk factor for final PROM scores and impactful on the magnitude of change among PROMs. Previous studies have investigated this relationship with the Simple Shoulder Test (SST), showing patients without diabetes with a higher initial SST score were more likely to have scored higher on their final SST (*P* < .05).^28^ Both their study, although more limited in patient population, and ours proved to show less favorable outcomes for those with higher patient-reported shoulder limitations at initial visit.^28^ Interestingly, sex, another nonmodifiable risk factor, was identified as a prognostic risk factor for a reduction in P-Interference and an increase in P-UE while also associated with a favorable change in P-UE and P-Interference. Like BMI and hyperlipidemia, this factor has been established as a risk factor for developing IAC, with up to 70% of patients with IAC being female.^32^ Loosely related, Candela et al’s study of 278 patients found there to be a significant difference between males and females with regards to initial pain intensity measured through VAS, favoring higher pain in females.^5^ However, contradictory to our finding, a smaller study (n = 47) conducted by Fernandes et al showed that female gender was independently associated with higher disability of the arm, shoulder, and hand scores (*P* = .0004).^16^

Our study does not come without limitations that must be considered. First, patient responses to surveys such as P-UE, P-Interference, P-Intensity, and VAS can vary over time due to recent events and a patient’s health status that are beyond the scope of the condition in question.^10^^,^^17^^,^^27^ Second, PROMs were administered in the same order for each patient which can introduce a level of survey burden. In an effort to control for this, further studies can introduce randomization of survey order. Third, our study was limited to only English-speaking patients. Accommodating for this limitation is possible; however, it would require the computer-adaptive surveys be translated into various languages and further studies be performed to ensure the internal validity of the surveys is retained after translation. Fourth, our study was limited to a single surgeon at a single institution which may limit generalizability.

## Conclusion

Patient-perceived improvements in PROMIS score during the natural history of adhesive capsulitis are likely multifactorial, with anxiety, hyperlipidemia, increased BMI, and Hispanic heritage associated with reduced improvement in PROMIS scores.

## Disclaimers

Funding: No outside funding or grants were received in support of the completion of this study.

Conflicts of interest: The authors, their immediate families, and any research foundation with which they are affiliated have not received any financial payments or other benefits from any commercial entity related to the subject of this article.
